# MicroRNA modulated networks of adaptive and innate immune response in pancreatic ductal adenocarcinoma

**DOI:** 10.1371/journal.pone.0217421

**Published:** 2019-05-31

**Authors:** Tainara F. Felix, Rainer M. Lopez Lapa, Márcio de Carvalho, Natália Bertoni, Tomas Tokar, Rogério A. Oliveira, Maria A. M. Rodrigues, Cláudia N. Hasimoto, Walmar K. Oliveira, Leonardo Pelafsky, César T. Spadella, Juan C. Llanos, Giovanni F. Silva, Wan L. Lam, Silvia Regina Rogatto, Luciana Schultz Amorim, Sandra A. Drigo, Robson F. Carvalho, Patricia P. Reis

**Affiliations:** 1 Department of Surgery and Orthopedics, Faculty of Medicine, São Paulo State University (UNESP), Botucatu, SP, Brazil; 2 Experimental Research Unity (UNIPEX), Faculty of Medicine, São Paulo State University (UNESP), Botucatu, SP, Brazil; 3 Department of Genetics, Institute of Biosciences, São Paulo State University (UNESP), Botucatu, SP, Brazil; 4 Department of Veterinary Clinic, School of Veterinary Medicine and Animal Science, São Paulo State University (UNESP), Botucatu, SP, Brazil; 5 Krembil Research Institute, University Health Network, Toronto, ON, Canada; 6 Department of Biostatistics, Institute of Biosciences, São Paulo State University (UNESP), Botucatu, SP, Brazil; 7 Department of Pathology, Faculty of Medicine, São Paulo State University (UNESP), Botucatu, SP, Brazil; 8 Department of Clinics and Gastroenterology, Faculty of Medicine, São Paulo State University (UNESP), Botucatu, SP, Brazil; 9 Genetics Unity, Integrative Oncology, British Columbia Cancer Center, Vancouver, BC, Canada; 10 Department of Clinical Genetics, Vejle Hospital, Institute of Regional Health Research, University of Southern Denmark, Denmark, DK; 11 Institute of Pathological Anatomy, Piracicaba, SP, Brazil; 12 Department of Morphology, Institute of Biosciences, São Paulo State University (UNESP), Botucatu, SP, Brazil; University of Nebraska Medical Center, UNITED STATES

## Abstract

Despite progress in treatment strategies, only ~24% of pancreatic ductal adenocarcinoma (PDAC) patients survive >1 year. Our goal was to elucidate deregulated pathways modulated by microRNAs (miRNAs) in PDAC and Vater ampulla (AMP) cancers. Global miRNA expression was identified in 19 PDAC, 6 AMP and 25 paired, histologically normal pancreatic tissues using the GeneChip 4.0 miRNA arrays. Computational approaches were used for miRNA target prediction/identification of miRNA-regulated pathways. Target gene expression was validated in 178 pancreatic cancer and 4 pancreatic normal tissues from The Cancer Genome Atlas (TCGA). 20 miRNAs were significantly deregulated (FC≥2 and *p*<0.05) (15 down- and 5 up-regulated) in PDAC. miR-216 family (miR-216a-3p, miR-216a-5p, miR-216b-3p and miR-216b-5p) was consistently down-regulated in PDAC. miRNA-modulated pathways are associated with innate and adaptive immune system responses in PDAC. AMP cancers showed 8 down- and 1 up-regulated miRNAs (FDR *p*<0.05). Most enriched pathways (*p*<0.01) were RAS and Nerve Growth Factor signaling. PDAC and AMP display different global miRNA expression profiles and miRNA regulated networks/tumorigenesis pathways. The immune response was enriched in PDAC, suggesting the existence of immune checkpoint pathways more relevant to PDAC than AMP.

## Introduction

Pancreatic cancer is the fourth most frequent cause of cancer death, worldwide [1,2]. The majority of pancreatic cancers (~96%) comprise exocrine ductal adenocarcinomas (PDAC) [2]. Other pancreatic tumors include periampullary carcinomas; of these, ~12% are adenocarcinomas of Vater ampulla (AMP). AMP patients have a better prognosis (5-year survival of >45%) [3,4] compared to PDAC, mainly due to early disease detection.

The asymptomatic nature of PDAC is often associated with late diagnosis and poor patient prognosis. This disease is characterized by very aggressive and rapid tumor growth and high incidence of distant metastasis [5]. Despite progress in treatment strategies (surgery, chemo and radiation therapies) in the past 5 years, only 24% of patients with PDAC survive more than a year and <5% are expected to survive more than 5 years after diagnosis [6]. More recently developed immunotherapy treatment strategies have failed to succeed for patients with PDAC. Therefore, there is a need to identify clinically useful biomarkers for early disease detection, as well as to further develop combinations of precision therapeutics for patients diagnosed with the late-stage disease [7].

Molecular mechanisms underlying pancreatic oncogenesis involve both genetic and epigenetic changes [8]. The role of microRNAs (miRNAs) has been widely reported as regulatory molecules associated with tumorigenesis [9]. miRNAs are potent gene expression regulators involved in biological processes including embryonic development, differentiation, apoptosis and cell proliferation [10,11]. An increasing number of non-coding RNAs, including miRNAs, have been reported to regulate immune system homeostasis and immune system development and function [12,13]. miRNAs have roles in the regulation of both innate and adaptive immune responses, in which they control early development of immune cell progenitors, maintenance and differentiation and mature immune cell function [12,14]. Considering that miRNAs have been shown as biomarkers for diagnosis, prognosis, and treatment of patients with cancer [15] they may have potential use as combination therapeutic molecules in PDAC, targeting immune system pathways.

Previous studies compared global miRNA expression profiles in PDAC and AMP and showed that miRNA expression profiles are distinct between PDAC and AMP tumors [16–20].

Here we sought to elucidate deregulated mechanisms modulated by miRNAs in PDAC and AMP cancers by experimental analysis followed by application of systems biology approaches. Our study shows that PDAC and AMP display different global miRNA expression profiles, which reflect distinct miRNA-regulated networks and pathways of tumorigenesis. Notably, an enrichment of immune response pathways was found in PDAC, suggesting that immune checkpoint pathways are more relevant to PDAC than AMP. The identification of miRNAs as biomarkers of innate and adaptive immune response in PDAC opens avenues for investigation of novel therapeutic strategies.

## Materials and methods

### Ethics statement

This study was performed in accordance with the national and international ethical guidelines and recommendations of the Declaration of Helsinki. This study was approved by the Research Ethics Committee of the Faculty of Medicine, UNESP, Botucatu, SP, Brazil (Protocol # 4382/2012-B).

### Patient samples

Formalin-fixed, paraffin-embedded (FFPE) tissue samples (paired tumors and histologically normal surgical resection margins) from patients undergoing surgery for resection of PDAC (N = 19) or AMP (N = 6) cancers were retrospectively collected from the Dept. of Pathology, Faculty of Medicine, UNESP, Botucatu, SP, Brazil. Primary pancreatic ductal adenocarcinoma and pancreatic carcinoma of the ampulla of Vater were obtained from patients naive of chemo or radiotherapy before surgery. Exclusion criteria were: patients diagnosed with secondary pancreatic metastasis and with unresectable disease. Patient demographic and histopathological data are shown in Table 1.

**Table 1:** Demographic and histopathological data of patients with PDAC and AMP.

Variables	PDAC N = 19	N (%)	AMP N = 6	N (%)
**Age (years)**				
**Median**	59		65	
**Average (range)**	59 (38–77)		60 (31 a 73)	
**Gender**				
**Male**	7	36.9	5	83.3
**Female**	12	63.1	1	16.7
**Tumor grade**				
**WD**	15	79	4	66.6
**MD**	2	10.5	1	16.7
**PD**	2	10.5	1	16.7
**Tumor size (T)**				
**T1-T2**	7	36.9	2	33.3
**T3-T4**	12	63.1	4	66.7
**Nodal status**				
**Positive (N1)**	13	68.5	2	33.3
**Negative (N0)**	2	10.5	4	66.7
**Undetermined (Nx)**	4	21	0	0
**Distant metastasis**				
**Positive (M1)**	0	0	0	0
**Negative (M0)**	0	0	0	0
**Undetermined (Mx)**	19	100	6	100
**Stage**				
**Ia**	1	5.25	2	33.3
**Ib**	0	0	0	0
**IIa**	1	5.25	2	33.3
**IIb**	17	89.5	2	33.3
**III**	0	0	0	0
**IV**	0	0	0	0

WD: well differentiated

MD: moderately differentiated

PD: poorly differentiated

### Tissue macrodissection and RNA extraction

Formalin-fixed, paraffin embedded (FFPE) tissue sections were obtained from surgically resected tumors (5–10 sections each, 10 μm thick). Tissue sections were prepared for needle macrodissection using the stereomicroscope Leica EZ4 (Leica Microsystems, Wetzlar, Germany), according to a previously reported protocol [21,22]. Tissue macrodissection was performed in order to isolate the total areas of the tumor including stromal cells, separate from adjacent, histologically normal pancreatic epithelia. A representative example of macrodissected areas (tumor and histologically normal pancreatic tissues) is shown in S1 Fig. Total RNA was then isolated from macrodissected samples and purified using the RecoverAll Total Nucleic Acid Isolation Kit (Ambion, Austin, TX, USA), according to the manufacturer’s protocol. This method allows isolation of total RNA, enriching for miRNAs from FFPE specimens. The protease digestion conditions of the RecoverAll kit are designed to release a maximal amount of RNA fragments of all sizes, including miRNAs. RNA quality and quantity were evaluated by spectrophotometry.

### Quantification of miRNA expression

GeneChip microRNA 4.0 array (Thermo Fisher Scientific, Waltham, MA, USA) containing probes representing 2,588 miRNAs (miRBase release 21; www.mirbase.org) was assayed according to the manufacturer´s protocol. Briefly, RNA samples were first subjected to poly-A tail incorporation to 3´-end, followed by a second step of ligation reaction and biotinylation of 3´-poly A tail of RNA. Biotin-labeled RNA was hybridized to the GeneChip miRNA 4.0 array cartridge, for each sample, and detected with Avidin-Streptavidin-Phycoerythrin (PE) conjugate, which binds to biotin-labeled RNA with strong affinity. miRNA array cartridges were then placed into hybridization oven trays and trays were loaded into the hybridization oven and incubated at 48°C with 60 rpm rotation for 16 hours. Upon hybridization, each array was filled with an array holding buffer and allowed to reach room temperature before washing and staining. Wash and stain steps were performed using the appropriate fluidics script for cartridge arrays on the fluidics station. Arrays were scanned and data exported for further analysis using the Expression Console software (Affymetrix) for data summarization, normalization, and quality control.

PDAC and AMP data were analyzed independently in order to identify significantly deregulated miRNAs (fold change, FC ≥ 2 and p<0.05) in each tumor subtype compared to their corresponding normal tissues. In order to decrease the small sample size effect of the AMP sample group (N = 6 tumors and 6 paired normal tissues), miRNA analysis for the AMP sample group did not rely in the *p*-values alone but utilized False Discovery Rate (FDR) *p*-values [23] to select miRNAs that are potentially relevant in AMP cancers.

Original, raw data are publicly available on Gene Expression Omnibus (GEO), under accession number GSE125179.

### miRNA target prediction analysis

Deregulated miRNAs were subjected to target prediction analysis using two different bioinformatic tools: microRNA Data Integration Portal (http://ophid.utoronto.ca/mirDIP/) [24], using as criteria for target selection, results with “very high” (top 1%) and “high” (top 5%) scores for interaction probability. In addition, we included validated interactions in miRTarBase (http://mirtarbase.mbc.nctu.edu.tw/php/index.php) [25].

*Validation of miRNA target gene expression in external data sources* (*TCGA*)

Next, predicted miRNA targets were validated using external data retrieved from The Cancer Genome Atlas (TCGA) (https://cancergenome.nih.gov/). Raw RNA sequencing (RNA-Seq) data (read counts) were used to calculate differential gene expression levels in pancreatic carcinoma samples (N = 178) relative to normal pancreatic tissues (N = 4). For AMP subtype, gene expression data were not available in TCGA (date of access: November 6^th^ 2018). We used edgeR package [26] in the default settings for gene expression analysis and generated a list of differentially expressed genes and corresponding fold change (FC) values. Genes were identified as over- or under-expressed with FDR≤0.05.

In addition, expression levels of miRNA target genes were analyzed using unsupervised clustering approaches, according to neoplastic cellularity of PDAC (high purity *vs*. low purity tumors). Gene expression data were analyzed using Xena Functional Genomics Explorer tool (https://xenabrowser.net/) [27]. Morpheus—Versatile matrix visualization and analysis software (https://software.broadinstitute.org/morpheus/) [28] was used to verify whether PDAC samples would cluster in separate groups according to the expression levels of the 86 genes and epithelial cellularity. Unsupervised hierarchical clustering was applied using Morpheus tool with the following parameters: Euclidean Distance, Average Data, Cluster Genes, and Samples. Significantly deregulated genes in pancreatic carcinoma, which are targeted by the identified miRNAs and with an inverse correlation with miRNA expression (e.g., miRNA under-expression / target gene over-expression) were used in subsequent pathway enrichment and network analyzes, as described below.

### Pathway enrichment and network analyses

ToppGene Suite (https://toppgene.cchmc.org/) [29] was used to identify statistically enriched pathways. Protein-protein interaction (PPI) network analysis was performed using Integrated Interactions Database (IID) (http://iid.ophid.utoronto.ca/iid/Search_By_Proteins/) [30] to assembly PPI networks between targets of differentially expressed miRNAs. Using IID, we obtained PPIs among the query genes, selecting for experimentally validated interactions. PPI networks were visualized using NAViGaTOR v.2.3.2 [31], which comprised target genes as the nodes, and PPIs among these genes as the edges.

**Fig 1:**
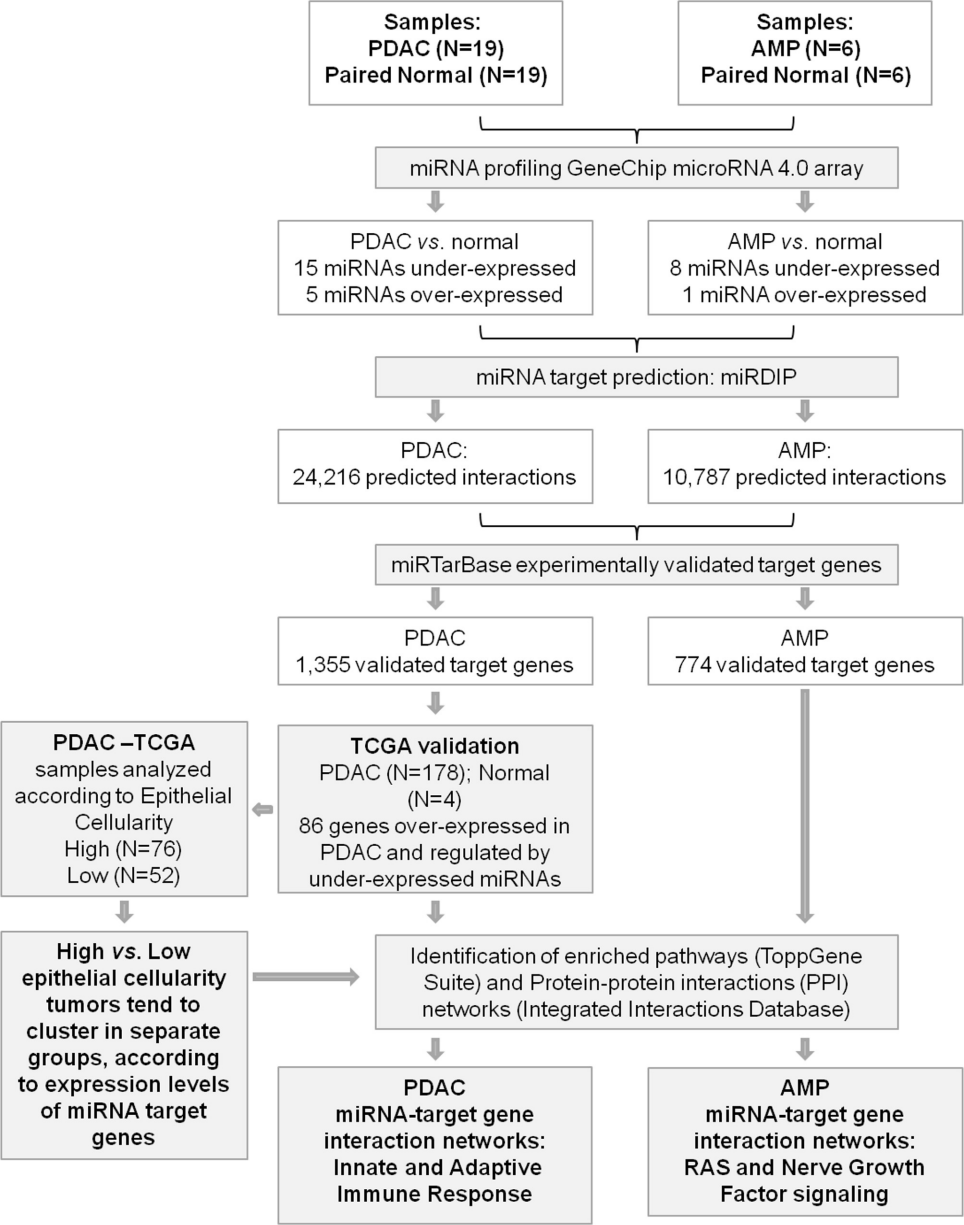
Flowchart integrating study design and main results.

Study design, methods of data analyses and results are shown in Fig 1.

## Results

### A subset of miRNAs is down-regulated and modulates validated gene targets within adaptive and innate immune response pathways in PDAC

**Fig 2:**
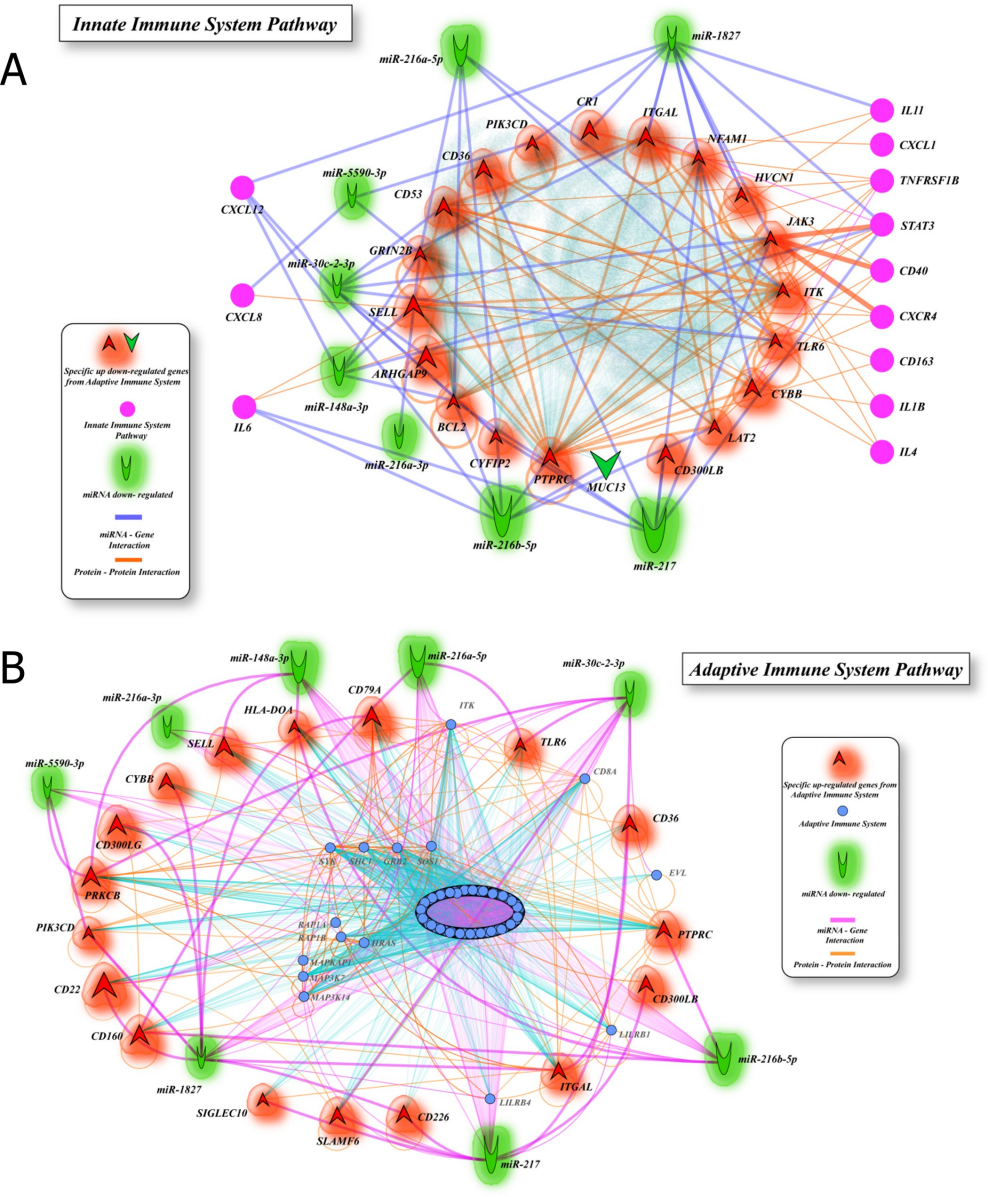
miRNA-gene interaction networks in PDAC for (a) innate and (b) adaptive immune system pathways. Genes found as significantly over-expressed (FC≥1.5 and p<0.05 and FDR≤0.05) in the pancreatic TCGA dataset are shown in red and labeled by their symbols, and are significantly enriched by under-expressed miRNAs (green). Genes labeled in pink are predicted miRNA targets and interact directly with over-expressed genes.

We identified 20 significantly deregulated miRNAs (FC≥2 and p<0.05) being 15 down-regulated and 5 up-regulated in PDAC compared to matched normal pancreatic tissues from the same patients (Table 2). Interestingly, miR-216 miRNA family (miR-216a-3p. miR-216a-5p, miR-216b-3p and miR-216b-5p) was identified as consistently down-regulated in PDAC. The highest differences were detected for under-expressed miRNAs: miR-217 (FC = -95.45), miR-216b-5p (FC = -62.86) and miR-148a-3p (FC = -33.75). Furthermore, mirDIP analysis with these 20 miRNAs revealed a total of 24,216 interactions filtered for “high” and “very high” interaction probability. Of these, 1,355 genes were validated targets in miRTarBase (S1 Table) and were further mapped on 16 significantly (p<0.05) enriched pathways. The most enriched pathways with a higher number of genes were the innate immune system response (1,312 genes) and adaptive immune response (826 genes) (S2 Fig). Additional pathways involved genes associated with mechanisms of the immune response regulation (S2 Table). miRNA-gene interaction networks of the innate and adaptive immune response are shown in Fig 2A and 2B. miRNA target genes with functions in the adaptive and innate immune system, as identified in our network analysis, were significantly over-expressed in the 178 PDAC samples from the TCGA dataset (Table 3).

**Table 2:** Deregulated miRNAs in PDAC compared to normal pancreatic tissues.

Down-regulated			Up-regulated		
MicroRNA	FC	p-value	microRNA	FC	p-value
hsa-miR-216a-5p	-52	0.000	hsa-miR-210-3p	10.05	0.017
hsa-miR-216a-3p	-5.82	0.000	hsa-miR-708-5p	9.17	0.029
hsa-miR-1827	-2.36	0.000	hsa-miR-106b-3p	4.2	0.031
hsa-miR-216b-3p	-2.14	0.000	hsa-miR-31-5p	12.89	0.04
hsa-miR-5590-3p	-2.09	0.000	hsa-miR-150-5p	7.02	0.047
hsa-miR-217	-95.45	0.001			
hsa-miR-216b-5p	-62.86	0.001			
hsa-miR-148a-3p	-33.75	0.001			
hsa-miR-30c-2-3p	-2.13	0.001			
hsa-miR-141-3p	-10.85	0.007			
hsa-miR-5195-5p	-2.2	0.008			
hsa-miR-130b-3p	-4.82	0.009			
hsa-miR-193b-5p	-2.62	0.015			
hsa-miR-6736-5p	-2.38	0.023			
hsa-miR-3163	-2.17	0.049			

FC: fold change. *p*-value: ANOVA test.

**Table 3:** Expression levels of genes involved in innate and/or adaptive immune system pathways, validated in the PDAC—TCGA dataset (N = 178 tumors and 4 normal tissues). Genes are over-expressed and predicted to be regulated by under-expressed miRNAs.

**Innate immune system pathway**			
Genes	FC	*p*-value	FDR
*CR1*	3.54	5.43E-11	5.22E-08
*ARHGAP9*	2.04	8.09E-06	0.000779105
*CD53*	1.97	1.08E-05	0.000931297
*HVCN1*	1.89	3.28E-06	0.000389503
*BCL2*	1.73	2.87E-05	0.002037914
*CYFIP2*	1.55	0.000245959	0.011148321
**Adaptive immune system pathway**			
Genes	FC	*p*-value	FDR
*CD22*	3.83	1.31E-10	1.13E-07
*CD160*	3.15	1.35E-23	1.10E-19
*CD79A*	2.71	0.000257143	0.011590682
*PRKCB*	2.64	2.34E-06	0.000302879
*CD300LG*	2.57	0.001662075	0.045301984
*SLAMF6*	2.14	5.96E-05	0.003631037
*PTPRC*	2.01	0.000187886	0.009208655
*SIGLEC10*	1.79	0.000156912	0.008054502
*CD226*	1.66	0.001735871	0.0470812
*HLA-DOA*	1.59	0.000543535	0.020331941
**Innate and Adaptive immune system pathways**			
Genes	FC	*p*-value	FDR
*CD36*	2.85	1.64E-08	7.18E-06
*ITGAL*	2.43	4.83E-07	9.37E-05
*CD300LB*	2.18	4.38E-07	8.69E-05
*SELL*	2.14	0.000300675	0.013081778
*TLR6*	1.73	4.17E-05	0.002772864
*PIK3CD*	1.67	8.46E-06	0.000805096
*CYBB*	1.67	0.000433473	0.017120097

FC = fold-change. FDR = false discovery rate.

### High and low epithelial cellularity PDAC samples tend to cluster in separate groups according to gene expression levels

**Fig 3:**
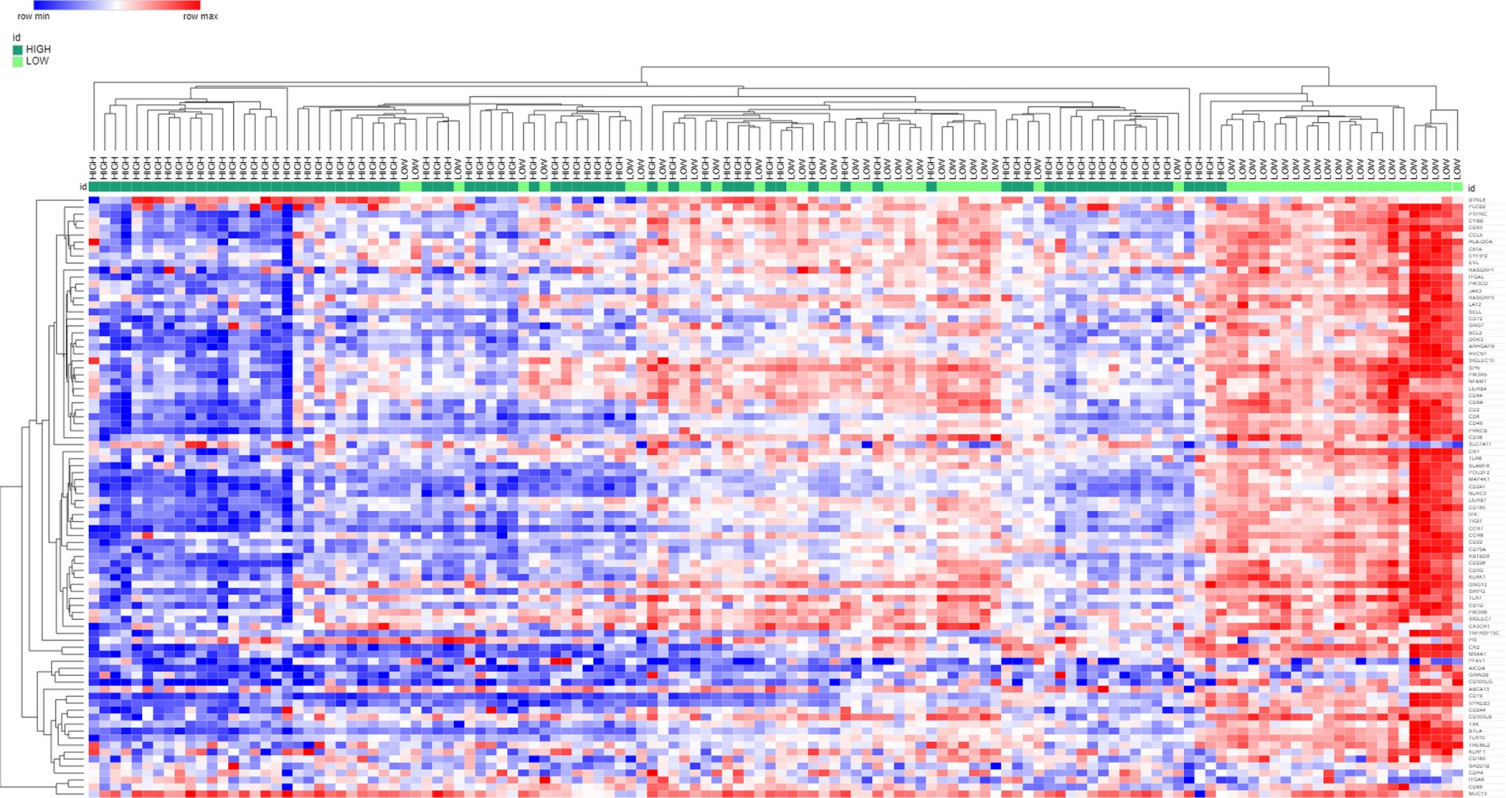
Heatmap showing unsupervised hierarchical clustering of high and low epithelial cellularity tumors according to gene expression levels (86 over-expressed genes regulated by under-expressed miRNAs). High and Low cellularity tumors tend to cluster in separate groups.

**Fig 4:**
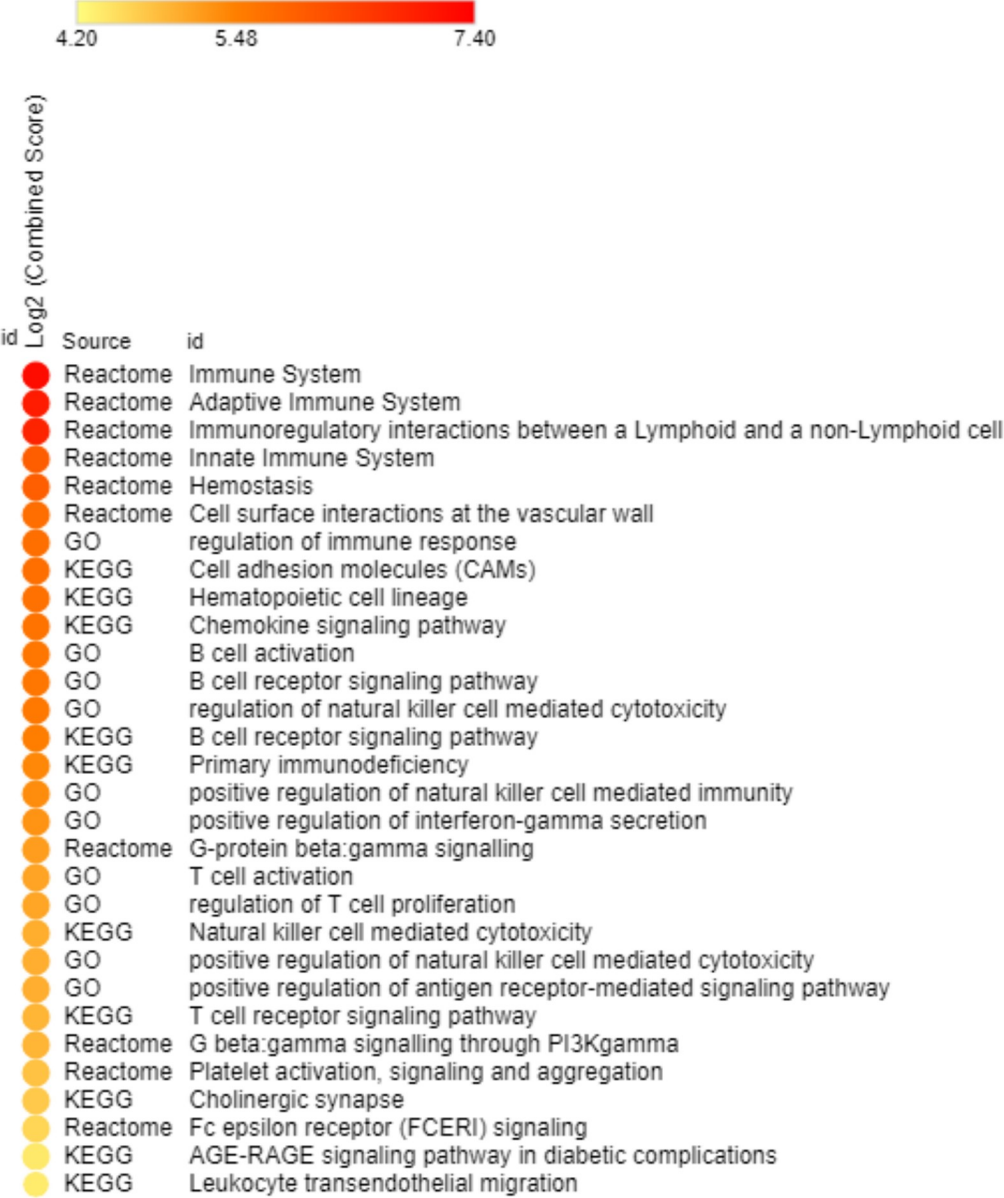
Enriched pathways for the 86 over-expressed genes targeted by under-expressed miRNAs in high and low epithelial cellularity PDAC samples.

Our comparative analyses stratifying the TCGA pancreatic tumor dataset into high and low epithelial cellularity tumors included 128 tumors histologically confirmed as pancreatic ductal adenocarcinoma and classified as High or Low cellularity tumors. Of the 128 PDAC tumors, 76 were High and 52 Low Purity/Cellularity samples (data extracted from TCGA). Expression levels of the 86 significantly deregulated genes in High *vs*. Low Cellularity tumors are shown in S3 Table. These genes were all over-expressed and targeted by the under-expressed microRNAs identified in our study. Unsupervised hierarchical clustering results showed that High and Low cellularity tumors tended to cluster in separate groups, with two enriched, smaller groups containing only High or Low cellularity tumors, and another group containing a mixture of these distinct classifications (Fig 3). In addition, enriched pathways for the 86 genes targeted by miRNAs and deregulated in the PDAC–TCGA dataset are shown in Fig 4.

### Deregulated miRNAs modulate target gene expression involved in cell growth and differentiation pathways in AMP

**Fig 5:**
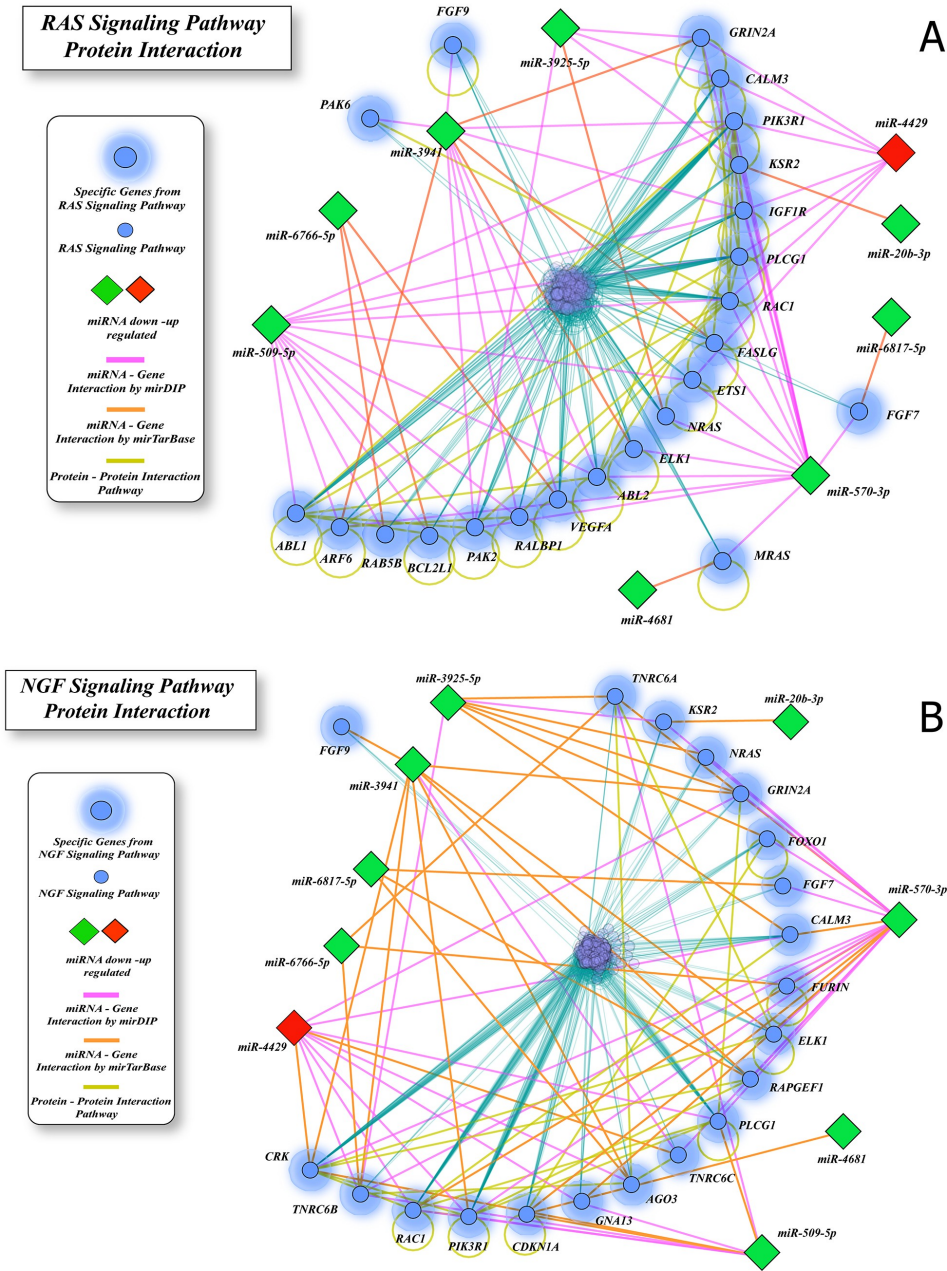
**miRNA-gene interaction network in AMP for (a) RAS signaling and (b) Nerve Growth Factor (NGF) signaling pathways.** Genes (shown in blue and labeled by their symbols) are significantly enriched by differentially expressed miRNAs (down-regulated: green; up-regulated: red).

We identified 9 miRNAs (8 under- and 1 over-expressed) as significantly differentially expressed (FDR p-value < 0.05) in AMP *vs*. normal tissues (Table 4). Target prediction analysis using mirDIP for these 9 miRNAs showed 10,787 interactions. Of these, 2,009 were validated interactions for 774 target genes on miRTarBase (S4 Table). These 774 genes were involved in 14 significantly (p≤0.01) enriched pathways (S5 Table). Of these, the most enriched pathways based on the number of involved genes were RAS and Nerve Growth Factor (NGF) signaling. miRNA-mRNA interaction networks for genes in these pathways are shown in Fig 5A and 5B.

**Table 4:** Deregulated miRNAs in adenocarcinoma of Vater ampulla (AMP).

**Under-expressed**		
**miRNA**	**FC**	**FDR *p*-value**
**hsa-miR-509-5p**	-2.27	0.012
**hsa-miR-4681**	-2.71	0.024
**hsa-mir-570**	-2.02	0.026
**hsa-miR-3925-5p**	-2.89	0.030
**hsa-miR-20b-3p**	-2.19	0.035
**hsa-miR-6817-5p**	-2.56	0.036
**hsa-miR-6766-5p**	-2.94	0.036
**hsa-miR-3941**	-2.28	0.040
**Over-expressed**		
**miRNA**	**FC**	**FDR *p*-value**
**hsa-miR-4429**	3.54	0.024

## Discussion

Recently studies have provided evidence of an immune-related component of PDAC tumors. Here, we provide novel data on the potential role of miRNAs in the regulation of immune-related gene networks including pathways of adaptive and innate immune response involved in PDAC. We validated an inverse correlation in miRNA and target gene expression in these pathways using the pancreatic cancer TCGA dataset; these data suggest a potential role of miRNAs in the regulation of immune-related genes in PDAC tumorigenesis.

Of note, our study used needle macrodissected samples including tumor cells and stromal cells, an important component of the tumor microenvironment. Another study [17] have also identified novel miRNAs associated with PDAC when analyzing tumor and stromal cells, without using any method of enrichment for tumor cells. The analysis of tumor and stromal cells allows the detection of miRNA expression profiles representative of tumor-stroma interactions. This is important, since invasive tumors such as PDAC have a characteristic, extensive desmoplastic stroma, which depends, at large, on regulatory signals to and from tumor cells. The desmoplastic stroma includes inflammation, neovascularization and activation of fibroblasts and cancer-associated fibroblasts, which actively participate in the tumor-stroma crosstalk, driving tumor development and progression [32,33].

Recently, it has been shown that the neoplastic cellularity of PDAC (high *vs*. low cellularity tumors) impacts the analyses and interpretation of transcriptomic data including coding and non-coding RNAs [34]. Results from our comparative analysis in high *vs*. low cellularity PDAC samples corroborate the current literature, showing that PDAC tumors are very complex and gene expression levels are associated with neoplastic epithelial cellularity in this carcinoma. Of note, our unsupervised hierarchical clustering results showed higher gene expression levels in low compared to high cellularity tumors. Since low cellularity tumors may contain tumor cells as well as stromal tissue; these data are in agreement with our miRNA data, which are reflective of miRNA alterations in tumor cells and stromal cells in PDAC.

Overall, our study identified a smaller number of deregulated miRNAs in PDAC, compared to one of the largest studies in PDAC [17]. Scientific and technical differences may explain these findings, such as the different number of patients, distinct platforms and methods of data analyses, and the nature of control samples used in both studies. Nevertheless, and more importantly, when we compared our data with the data by Schultz *et al*. [17], we observed that 6 miRNAs (miR-130b-3p, miR-141-3p, miR-148a, miR-216a, miR-216b, and miR-217) were commonly under-expressed in both studies. Considering that patients in both studies were from different geographical locations, we highlight this commonly altered subset of miRNAs as relevant for PDAC.

Recently, integrative transcriptional profiling analysis of primary PDAC, pancreatic human and mouse cell lines defined three PDAC subtypes: classical, quasimesenchymal and exocrine-like, which show differences in outcome and therapeutic responses [35]. In another study [36], basal-like and classical pancreatic tumor subtypes were identified. Additionally, an immunogenic subtype of pancreatic cancer has been described [37], in which tumors harbor upregulated immune networks and distinct molecular pathways that may explain the evolution of this different disease subtype. An immune stromal component was validated in PDAC, containing a high expression of CD37, CD53, CD4, and CSF1R [38]. We validated CD53 over-expression in the pancreatic TCGA dataset. CD53 is targeted by miR-30c, which is under-expressed in our PDAC dataset.

PDAC has a characteristic complexity of inflammatory and regulatory immune cells in tumor cell infiltrates in crosstalk with the tumor microenvironment, which is composed of a variety of immune cells [39,40]. In the tumor microenvironment, cytokine production can promote cancer cell growth [41]. We found a number of cytokines regulated by miRNAs in innate immunity, including IL-6, which is a direct target of miRNAs miR-216-5p and miR-217, CXCL8/IL-8, targeted by miR-5590-3p, IL-11 regulated by miR-1827. Under-expression of the miR-216 cluster (miR-216a-3p, miR-216a-5p, miR-216b-3p and miR-216b-5p) was consistently observed in our PDAC samples compared to normal pancreatic tissues. Significant reduction in expression of the miR-216 cluster has been reported in pancreatic cancer and induced expression of these miRNAs decreased tumor cell aggressiveness, indicating that these miRNAs have a tumor suppressor effect [42].

In pancreatic cancer, high expression levels of IL-6, a cytokine produced by a variety of cells including immune and pancreatic tumor cells [43], and IL-1β were correlated with poor overall and progression-free survival in pancreatic cancer patients [44]. IL-6 gene is strongly over-expressed in pancreatic cancer compared to normal tissues [45,46] and IL-6 protein has strong cytoplasm expression in pancreatic tumor cells [47]. IL-6 is also highly expressed in serum from PDAC patients compared to non-diseased individuals and associated with disease aggressiveness and inflammatory response [48] and pancreatic cancer-related cachexia [47]. In our network analysis, we show miRNA-gene interactions highlighting known mechanisms such as the signal transduction activated by IL-6 that in turn activate JAK2 and STAT3. Importantly, a recent study demonstrated that silencing of IL-6 leads to increased sensitivity to gemcitabine treatment [49].

IL-1β, IL-4, and IL-8 interact with other miRNA-regulated targets within the innate immune response network. These cytokines in addition to VEGF, TGF, and IL-10 were consistently over-expressed in different sources of samples from PDAC patients [50]. CXCL-12 production is regulated by a number of miRNAs found under-expressed in our PDAC data: miR-30c-2-3p, miR-148a-3p, miR-216b-5p and miR-1827. CXCR4 was predicted to have a strong interaction with JAK3, which is regulated by miR-30c-2-3p and miR-217, which are under-expressed in PDAC.

The adaptive immune response pathway was correlated with a number of down-regulated miRNAs in our PDAC dataset: 8/20 (40%) miRNAs identified in our study directly target genes in this pathway. Specifically, members of miR-216 family (miR-216a-5p, miR-216a-3p and miR-216b-5p) and miR-217 target CD antigens including LILRB1 and LILRB4 [51], CD36, CD160 [52], CD226 [53], CD300LB and SIGLEC10 [54] as well as toll-like receptor TLR6. In the context of the tumor microenvironment, gene expression alterations may be dependent on post-transcriptional regulatory mechanisms such as miRNAs, which have been shown to regulate the recruitment and activation of immune cells to the tumor [12].

Considering that the PDAC tissues contain a variety of cells including tumor cells, inflammatory cells, fibroblasts, we searched for the specific cellular localization of miRNAs. For this, we used the Functional Annotation of Mammalian Genome (FANTOM5) consortium data. This consortium provides small RNA sequencing data on 396 human samples across 118 cell types obtained from different organs [55]. We searched this database for all miRNAs found as significantly under-expressed in tumor *vs*. normal (Table 2). The results showed enriched Gene Ontologies for a subset of miRNAs: miR-216a-3p, miR-216b-5p, miR-217 and miR-130b-3p are mainly expressed in endothelial cells of vascular tree; miR-141-3p and miR-3163 are mainly expressed by epithelial cells, and miR-148a is mainly expressed by mast cells, myeloid leukocytes, and secretory cells. Of note, mast cells are present in the tumor stroma, contributing to the inflammatory microenvironment that impacts tumor behavior [56,57]. In the context of our findings, mir-148a under-expression in tumor and stroma potentially leads to increased expression of its target genes such as *BCL2*, *GRIN2B*, and *SELL*, which play roles including adaptive immune system regulation. Recent studies showed that tumor cells are capable of evading immune response by increasing the expression of genes that encode inhibitory molecules or adhesion molecules. Interestingly, *SELL* over-expression has been associated with differential immune cell infiltration in lung cancer [58]. In pancreatic ductal adenocarcinoma, *ITGAL*, which encodes another adhesion molecule and is also shown in our miRNA-mRNA networks, was over-expressed in tumor cells. Interestingly, *ITGAL* has been associated with enriched pathways of granulocyte adhesion and diapedesis and leukocyte extravasation signaling [59]. In conclusion, the identified miRNAs may play important roles in tumor cells modulating important oncogenic pathways being reflective of tumor-microenvironment interactions, particularly by modulating immune tumor response.

In AMP cancers, we highlight RAS, NGF and EGFR and axon guidance signaling pathways, since identified miRNAs modulate the expression of target genes with overlapping roles in all three pathways. Altered expression of genes in these pathways is associated with deregulated expression of 9 miRNAs: over-expression of miR-4429 and under-expression of miRNAs miR-20b-3p,miR-509-5p, miR-570-3p, miR-3925-5p, miR-3941, miR-4681, miR-6766-5p, and miR-6817-5p.

Interestingly, miR-323a-3p was also reported as over-expressed in pancreatic ampullary tumors and found to participate in interaction networks, modulating gene expression including *KRAS*, with roles in axon guidance pathway [60]. Results from this and our study suggest alternative miRNA regulatory networks modulating similar pathways in AMP cancers. Additionally, the NGF pathway has been previously reported to be induced by inflammatory cytokines in experimental models of liver disease [61–63]. NGF has been shown to alter the expression of several genes implicated in ovarian oncogenesis and a potential mechanism of NGF-regulated protein synthesis is by post-transcriptional miRNA regulation [64]. Notably, axon guidance, NGF and EGFR signaling mechanisms share a number of genes that have been already demonstrated to play important roles in tumor development and progression, such as the regulatory subunits of the phosphoinositide 3-kinases, PI3Ks, a family of lipid kinases that coordinate several cellular functions including proliferation and survival and are deregulated in different cancer types, including pancreatic cancer [65,66]. Genes within the EGFR signaling pathway have been shown to be frequently altered at both genomic and protein levels, contributing to tumor growth and progression of ampullary pancreatic carcinoma [67]. Axon guidance has been reported in the regulation of numerous biological processes including cancer [54,68]. Genes regulating axon guidance were frequently mutated and showed copy number alterations in PDAC at early stages [69]. The axon guidance factor netrin-1 has been shown to mediate effects of *MUC4* gene in neural invasion in PDAC [70]. Considering that our subset of patients with AMP had early-stage tumors, these data suggest that axon guidance pathways may be involved in early tumor development in different histological subtypes of pancreatic carcinoma.

In conclusion, our study identified miRNA changes in tumor and stromal cells of PDAC and AMP; such changes likely contribute to modulate the translational efficiency of corresponding target genes; however, we highlight that there are other mechanisms that interplay for translational regulation influencing gene expression. Indeed, translational control of gene expression occurs through a highly complex and dynamic interplay between mRNA sequences (cis-regulators) and the translational machinery, or canonical translational factors [71]. In addition to these factors, miRNAs are among the trans-regulator molecules that interact with the translational machinery core, to modulate translation of mRNA [Reviewed in [72]].

In addition, while functional studies may lead to a deeper understanding on the role of identified miRNAs on specific pathways, the coordinated functions of multiple miRNAs and their targets into regulatory networks have yet to be elucidated in the different subtypes of pancreatic cancer.

Our results suggest that miRNA-modulated pathways of the adaptive and innate immune response may represent an opportunity for improved therapeutics to benefit patients with pancreatic ductal adenocarcinoma.

## Conclusions

Our data provide a comprehensive picture of distinct pathways modulated by miRNAs in different subtypes of pancreatic tumors. Adaptive and innate immune response pathways modulated by miRNAs may be useful as potential therapeutic strategies specifically benefiting patients with PDAC.

## Supporting information

S1 Fig Representative example of macrodissected areas: Hematoxylin and Eosin (H&E) stained section showing in (A) adjacent normal tissue; normal pancreatic ducts are shown by the arrows and (B) pancreatic ductal adenocarcinoma cells and stromal component. Magnification: 200 μm.
(TIF)

S2 Fig Pathways with a higher number of miRNA-target genes in PDAC were the innate and adaptive immune responses. Graphical representation of miRNA target genes (Y-axis) according to significantly enriched pathways (p<0.05) (X-axis).
(TIF)

S3 Fig Pathways with a higher number of miRNA-target genes in AMP tumors were RAS and Nerve Growth Factor (NGF) signaling. Graphical representation of miRNA target genes (Y-axis) according to significantly enriched pathways (p<0.01) (X-axis).
(TIF)

S1 Table Predicted miRNA target genes in PDAC, validated in miRTarBase. (XLSX)

S2 Table miRNA modulated pathways in PDAC. (XLSX)

S3 Table Expression levels of 86 deregulated genes in PDAC stratifying by epithelial cellularity. (XLSX)

S4 Table Predicted miRNA target genes in AMP, validated in miRTarBase. (XLSX)

S5 Table miRNA modulated pathways in AMP. (XLSX)

## References

[1] Ferlay J, Colombet M, Soerjomataram I, Mathers C, Parkin DM, Piñeros M, et al Estimating the global cancer incidence and mortality in 2018: GLOBOCAN sources and methods. Int J Cancer. 2018; 10.1002/ijc.31937 30350310

[2] Siegel RL, Miller KD, Jemal A. Cancer statistics, 2018. CA Cancer J Clin. 2018;68: 7–30. 10.3322/caac.21442 29313949

[3] Askew J, Connor S. Review of the investigation and surgical management of resectable ampullary adenocarcinoma. HPB. 2013;15: 829–838. 10.1111/hpb.12038 23458317 PMC4503279

[4] Ahn DH, Bekaii-Saab T. Ampullary cancer: an overview. Am Soc Clin Oncol Educ B. 2014; 112–115. 10.14694/EdBook_AM.2014.34.112 24857067 PMC4966534

[5] Cid-Arregui A, Juarez V. Perspectives in the treatment of pancreatic adenocarcinoma. World J Gastroenterol. 2015;21: 9297–9316. 10.3748/wjg.v21.i31.9297 26309356 PMC4541382

[6] DeVita VTJ, Lawrence TS, Rosenberg SA. Cancer of the Pancreas. Cancer Principles & Pratice of Oncology. 2011 pp. 961–989.

[7] Morrison AH, Byrne KT, Vonderheide RH. Immunotherapy and Prevention of Pancreatic Cancer. Trends Cancer. 2018;4: 418–428. 10.1016/j.trecan.2018.04.001 29860986 PMC6028935

[8] Birnbaum DJ, Bertucci F, Finetti P, Birnbaum D, Mamessier E. Molecular classification as prognostic factor and guide for treatment decision of pancreatic cancer. Biochim Biophys Acta Rev Cancer. 2018;1869: 248–255. 10.1016/j.bbcan.2018.02.001 29499330

[9] Rupaimoole R, Slack FJ. MicroRNA therapeutics: towards a new era for the management of cancer and other diseases. Nat Rev Drug Discov. 2017;16: 203–222. 10.1038/nrd.2016.246 28209991

[10] Peng Y, Croce CM. The role of MicroRNAs in human cancer. Signal Transduct Target Ther. 2016;1: 15004 10.1038/sigtrans.2015.4 29263891 PMC5661652

[11] Nugent M. MicroRNAs and Fracture Healing. Calcif Tissue Int. 2017;101: 355–361. 10.1007/s00223-017-0296-x 28589206

[12] Curtale G, Citarella F. Dynamic nature of noncoding RNA regulation of adaptive immune response. Int J Mol Sci. 2013;14: 17347–17377. 10.3390/ijms140917347 23975170 PMC3794731

[13] Mehta A, Baltimore D. MicroRNAs as regulatory elements in immune system logic. Nat Rev Immunol. 2016;16: 279–294. 10.1038/nri.2016.40 27121651

[14] Paladini L, Fabris L, Bottai G, Raschioni C, Calin GA, Santarpia L. Targeting microRNAs as key modulators of tumor immune response. J Exp Clin Cancer Res. 2016;35: 103 10.1186/s13046-016-0375-2 27349385 PMC4924278

[15] Yuan HL, Wang T, Zhang KH. MicroRNAs as potential biomarkers for diagnosis, therapy and prognosis of gastric cancer. Onco Targets Ther. 2018;11: 3891–3900. 10.2147/OTT.S156921 30013369 PMC6039071

[16] Mazza T, Copetti M, Capocefalo D, Fusilli C, Biagini T, Carella M, et al MicroRNA co-expression networks exhibit increased complexity in pancreatic ductal compared to Vater’s papilla adenocarcinoma. Oncotarget. 2017;8: 105320–105339. 10.18632/oncotarget.22184 29285254 PMC5739641

[17] Schultz NA, Werner J, Willenbrock H, Roslind A, Giese N, Horn T, et al MicroRNA expression profiles associated with pancreatic adenocarcinoma and ampullary adenocarcinoma. Mod Pathol. 2012;25: 1609–1622. 10.1038/modpathol.2012.122 22878649

[18] Lee EJ, Gusev Y, Jiang J, Nuovo GJ, Lerner MR, Frankel WL, et al Expression profiling identifies microRNA signature in pancreatic cancer. Int J Cancer. 2007;120: 1046–1054. 10.1002/ijc.22394 17149698 PMC2680248

[19] Bloomston M, Frankel WL, Petrocca F, Volinia S, Alder H, Hagan JP, et al MicroRNA expression patterns to differentiate pancreatic adenocarcinoma from normal pancreas and chronic pancreatitis. JAMA. 2007;297: 1901–1908. 10.1001/jama.297.17.1901 17473300

[20] Szafranska AE, Davison TS, John J, Cannon T, Sipos B, Maghnouj A, et al MicroRNA expression alterations are linked to tumorigenesis and non-neoplastic processes in pancreatic ductal adenocarcinoma. Oncogene. 2007;26: 4442–4452. 10.1038/sj.onc.1210228 17237814

[21] Cervigne NK, Reis PP, Machado J, Sadikovic B, Bradley G, Galloni NN, et al Identification of a microRNA signature associated with progression of leukoplakia to oral carcinoma. Hum Mol Genet. Narnia; 2009;18: 4818–4829. 10.1093/hmg/ddp446 19776030

[22] Cervigne NK, Machado J, Goswami RS, Sadikovic B, Bradley G, Perez-Ordonez B, et al Recurrent genomic alterations in sequential progressive leukoplakia and oral cancer: drivers of oral tumorigenesis? Hum Mol Genet. Narnia; 2014;23: 2618–2628. 10.1093/hmg/ddt657 24403051 PMC3990162

[23] Forstmeier W, Wagenmakers E-J, Parker TH. Detecting and avoiding likely false-positive findings - a practical guide. Biol Rev. 2017;92: 1941–1968. 10.1111/brv.12315 27879038

[24] Tokar T, Pastrello C, Rossos AEM, Abovsky M, Hauschild AC, Tsay M, et al mirDIP 4.1-integrative database of human microRNA target predictions. Nucleic Acids Res. 2018;46: D360–D370. 10.1093/nar/gkx1144 29194489 PMC5753284

[25] Chou CH, Shrestha S, Yang CD, Chang NW, Lin YL, Liao KW, et al miRTarBase update 2018: a resource for experimentally validated microRNA-target interactions. Nucleic Acids Res. 2018;46: D296–D302. 10.1093/nar/gkx1067 29126174 PMC5753222

[26] Robinson MD, McCarthy DJ, Smyth GK. edgeR: a Bioconductor package for differential expression analysis of digital gene expression data. Bioinformatics. 2010;26: 139–140. 10.1093/bioinformatics/btp616 19910308 PMC2796818

[27] Goldman M, Craft B, Kamath A, Brooks AN, Zhu J, Haussler D. The UCSC Xena Platform for cancer genomics data visualization and interpretation. bioRxiv. Cold Spring Harbor Laboratory; 2018; 326470 10.1101/326470

[28] Starruß J, de Back W, Brusch L, Deutsch A. Morpheus: a user-friendly modeling environment for multiscale and multicellular systems biology. Bioinformatics. 2014;30: 1331–1332. 10.1093/bioinformatics/btt772 24443380 PMC3998129

[29] Chen J, Bardes EE, Aronow BJ, Jegga AG. ToppGene Suite for gene list enrichment analysis and candidate gene prioritization. Nucleic Acids Res. 2009;37: W305–11. 10.1093/nar/gkp427 19465376 PMC2703978

[30] Kotlyar M, Pastrello C, Sheahan N, Jurisica I. Integrated interactions database: tissue-specific view of the human and model organism interactomes. Nucleic Acids Res. 2016;44: D536–41. 10.1093/nar/gkv1115 26516188 PMC4702811

[31] Brown KR, Otasek D, Ali M, McGuffin MJ, Xie W, Devani B, et al NAViGaTOR: Network Analysis, Visualization and Graphing Toronto. Bioinformatics. 2009;25: 3327–3329. 10.1093/bioinformatics/btp595 19837718 PMC2788933

[32] Chu GC, Kimmelman AC, Hezel AF, DePinho RA. Stromal biology of pancreatic cancer. J Cell Biochem. 2007;101: 887–907. 10.1002/jcb.21209 17266048

[33] Thomas D, Radhakrishnan P. Tumor-stromal crosstalk in pancreatic cancer and tissue fibrosis. Mol Cancer. BioMed Central; 2019;18: 14 10.1186/s12943-018-0927-5 30665410 PMC6341551

[34] Raphael BJ, Hruban RH, Aguirre AJ, Moffitt RA, Yeh JJ, Stewart C, et al Integrated Genomic Characterization of Pancreatic Ductal Adenocarcinoma. Cancer Cell. 2017;32: 185–203.e13. 10.1016/j.ccell.2017.07.007 28810144 PMC5964983

[35] Collisson EA, Sadanandam A, Olson P, Gibb WJ, Truitt M, Gu S, et al Subtypes of pancreatic ductal adenocarcinoma and their differing responses to therapy. Nat Med. Nature Publishing Group; 2011;17: 500–503. 10.1038/nm.2344 21460848 PMC3755490

[36] Moffitt RA, Marayati R, Flate EL, Volmar KE, Loeza SGH, Hoadley KA, et al Virtual microdissection identifies distinct tumor- and stroma-specific subtypes of pancreatic ductal adenocarcinoma. Nat Genet. 2015;47: 1168–1178. 10.1038/ng.3398 26343385 PMC4912058

[37] Bailey P, Chang DK, Nones K, Johns AL, Patch AM, Gingras MC, et al Genomic analyses identify molecular subtypes of pancreatic cancer. Nature. Nature Publishing Group; 2016;531: 47–52. 10.1038/nature16965 26909576

[38] Puleo F, Nicolle R, Blum Y, Cros J, Marisa L, Demetter P, et al Stratification of Pancreatic Ductal Adenocarcinomas Based on Tumor and Microenvironment Features. Gastroenterology. 2018;155: 1999–2013.e3. 10.1053/j.gastro.2018.08.033 30165049

[39] Martinez-Bosch N, Vinaixa J, Navarro P. Immune Evasion in Pancreatic Cancer: From Mechanisms to Therapy. Cancers (Basel). 2018;10 10.3390/cancers10010006 29301364 PMC5789356

[40] Shibuya KC, Goel VK, Xiong W, Sham JG, Pollack SM, Leahy AM, et al Pancreatic ductal adenocarcinoma contains an effector and regulatory immune cell infiltrate that is altered by multimodal neoadjuvant treatment. PLoS One. 2014;9: e96565 10.1371/journal.pone.0096565 24794217 PMC4008589

[41] Wörmann SM, Diakopoulos KN, Lesina M, Algül H. The immune network in pancreatic cancer development and progression. Oncogene. 2014;33: 2956–2967. 10.1038/onc.2013.257 23851493

[42] Yonemori K, Seki N, Idichi T, Kurahara H, Osako Y, Koshizuka K, et al The microRNA expression signature of pancreatic ductal adenocarcinoma by RNA sequencing: anti-tumour functions of the microRNA-216 cluster. Oncotarget. 2017;8: 70097–70115. 10.18632/oncotarget.19591 29050264 PMC5642539

[43] Van Snick J. Interleukin-6: an overview. Annu Rev Immunol. 1990;8: 253–278. 10.1146/annurev.iy.08.040190.001345 2188664

[44] Mitsunaga S, Ikeda M, Shimizu S, Ohno I, Furuse J, Inagaki M, et al Serum levels of IL-6 and IL-1β can predict the efficacy of gemcitabine in patients with advanced pancreatic cancer. Br J Cancer. 2013;108: 2063–2069. 10.1038/bjc.2013.174 23591198 PMC3670479

[45] Bellone G, Smirne C, Mauri FA, Tonel E, Carbone A, Buffolino A, et al Cytokine expression profile in human pancreatic carcinoma cells and in surgical specimens: implications for survival. Cancer Immunol Immunother. 2006;55: 684–698. 10.1007/s00262-005-0047-0 16094523 PMC11031060

[46] Xing HB, Tong MT, Wang J, Hu H, Zhai CY, Huang CX, et al Suppression of. Biomed Res Int. 2018;2018: 3195025 10.1155/2018/3195025 29693005 PMC5859857

[47] Martignoni ME, Kunze P, Hildebrandt W, Künzli B, Berberat P, Giese T, et al Role of mononuclear cells and inflammatory cytokines in pancreatic cancer-related cachexia. Clin Cancer Res. 2005;11: 5802–5808. 10.1158/1078-0432.CCR-05-0185 16115919

[48] Okada S, Okusaka T, Ishii H, Kyogoku A, Yoshimori M, Kajimura N, et al Elevated serum interleukin-6 levels in patients with pancreatic cancer. Jpn J Clin Oncol. 1998;28: 12–15. Available: https://www.ncbi.nlm.nih.gov/pubmed/9491135 10.1093/jjco/28.1.12 9491135

[49] Xing HB, Tong MT, Wang J, Hu H, Zhai CY, Huang CX, et al Suppression of IL-6 gene by shRNA augments gemcitabine chemosensitization in pancreatic adenocarcinoma cells. Biomed Res Int. 2018;2018. 10.1155/2018/3195025 29693005 PMC5859857

[50] Yako YY, Kruger D, Smith M, Brand M. Cytokines as Biomarkers of Pancreatic Ductal Adenocarcinoma: A Systematic Review. PLoS One. 2016;11: e0154016 10.1371/journal.pone.0154016 27170998 PMC4865360

[51] Cortesini R. Pancreas cancer and the role of soluble immunoglobulin-like transcript 3 (ILT3). JOP. 2007;8: 697–703. Available: https://www.ncbi.nlm.nih.gov/pubmed/17993722 17993722

[52] Tu TC, Brown NK, Kim TJ, Wroblewska J, Yang X, Guo X, et al CD160 is essential for NK-mediated IFN-γ production. J Exp Med. 2015;212: 415–429. 10.1084/jem.20131601 25711213 PMC4354368

[53] Pauken KE, Wherry EJ. TIGIT and CD226: tipping the balance between costimulatory and coinhibitory molecules to augment the cancer immunotherapy toolkit. Cancer Cell. 2014;26: 785–787. 10.1016/j.ccell.2014.11.016 25490444

[54] Zhang L, Zhang Y, Wong SH, Law PTY, Zhao S, Yu J, et al Common Deregulation of Seven Biological Processes by MicroRNAs in Gastrointestinal Cancers. Sci Rep. 2018;8: 3287 10.1038/s41598-018-21573-w 29459716 PMC5818544

[55] de Rie D, Abugessaisa I, Alam T, Arner E, Arner P, Ashoor H, et al An integrated expression atlas of miRNAs and their promoters in human and mouse. Nat Biotechnol. 2017;35: 872–878. 10.1038/nbt.3947 28829439 PMC5767576

[56] Galdiero MR, Garlanda C, Jaillon S, Marone G, Mantovani A. Tumor associated macrophages and neutrophils in tumor progression. J Cell Physiol. 2013;228: 1404–1412. 10.1002/jcp.24260 23065796

[57] Varricchi G, Galdiero MR, Loffredo S, Marone G, Iannone R, Marone G, et al Are Mast Cells MASTers in Cancer? Front Immunol. Frontiers; 2017;8: 424 10.3389/fimmu.2017.00424 28446910 PMC5388770

[58] Chae YK, Choi WM, Bae WH, Anker J, Davis AA, Agte S, et al Overexpression of adhesion molecules and barrier molecules is associated with differential infiltration of immune cells in non-small cell lung cancer. Sci Rep. Nature Publishing Group; 2018;8: 1023 10.1038/s41598-018-19454-3 29348685 PMC5773521

[59] Mao Y, Shen J, Lu Y, Lin K, Wang H, Li Y, et al RNA sequencing analyses reveal novel differentially expressed genes and pathways in pancreatic cancer. Oncotarget. 2017;8: 42537–42547. 10.18632/oncotarget.16451 28418924 PMC5522086

[60] Mazza T, Copetti M, Capocefalo D, Fusilli C, Biagini T, Carella M, et al MicroRNA co-expression networks exhibit increased complexity in pancreatic ductal compared to Vater’s papilla adenocarcinoma. Oncotarget. 2017;8: 105320–105339. 10.18632/oncotarget.22184 29285254 PMC5739641

[61] Oakley F, Trim N, Constandinou CM, Ye W, Gray AM, Frantz G, et al Hepatocytes express nerve growth factor during liver injury: evidence for paracrine regulation of hepatic stellate cell apoptosis. Am J Pathol. 2003;163: 1849–1858. 10.1016/S0002-9440(10)63544-4 14578185 PMC1892444

[62] Gigliozzi A, Alpini G, Baroni GS, Marucci L, Metalli VD, Glaser SS, et al Nerve growth factor modulates the proliferative capacity of the intrahepatic biliary epithelium in experimental cholestasis. Gastroenterology. 2004;127: 1198–1209. Available: https://www.ncbi.nlm.nih.gov/pubmed/15480997 15480997 10.1053/j.gastro.2004.06.023

[63] Kao YH, Chen CL, Jawan B, Chung YH, Sun CK, Kuo SM, et al Upregulation of hepatoma-derived growth factor is involved in murine hepatic fibrogenesis. J Hepatol. 2010;52: 96–105. 10.1016/j.jhep.2009.10.002 19913322

[64] Retamales-Ortega R, Oróstica L, Vera C, Cuevas P, Hernández A, Hurtado I, et al Role of nerve growth factor (NGF) and miRNAs in epithelial ovarian cancer. Int J Mol Sci. 2017;18 10.3390/ijms18030507 28245631 PMC5372523

[65] Zhou L, Baba Y, Kitano Y, Miyake K, Zhang X, Yamamura K, et al KRAS, BRAF, and PIK3CA mutations, and patient prognosis in 126 pancreatic cancers: pyrosequencing technology and literature review. Med Oncol. 2016;33: 32 10.1007/s12032-016-0745-9 26927447

[66] Wang J, Paris PL, Chen J, Ngo V, Yao H, Frazier ML, et al Next generation sequencing of pancreatic cyst fluid microRNAs from low grade-benign and high grade-invasive lesions. Cancer Lett. 2015;356: 404–409. 10.1016/j.canlet.2014.09.029 25304377 PMC6200344

[67] Mikhitarian K, Pollen M, Zhao Z, Shyr Y, Merchant NB, Parikh A, et al Epidermal growth factor receptor signaling pathway is frequently altered in ampullary carcinoma at protein and genetic levels. Mod Pathol. 2014;27: 665–674. 10.1038/modpathol.2013.185 24186143 PMC4007414

[68] Kellermeyer R, Heydman LM, Mastick GS, Kidd T. The Role of Apoptotic Signaling in Axon Guidance. J Dev Biol. 2018;6 10.3390/jdb6040024 30340315 PMC6316149

[69] Biankin A V, Waddell N, Kassahn KS, Gingras MC, Muthuswamy LB, Johns AL, et al Pancreatic cancer genomes reveal aberrations in axon guidance pathway genes. Nature. 2012;491: 399–405. 10.1038/nature11547 23103869 PMC3530898

[70] Wang L, Zhi X, Zhu Y, Zhang Q, Wang W, Li Z, et al MUC4-promoted neural invasion is mediated by the axon guidance factor Netrin-1 in PDAC. Oncotarget. 2015;6: 33805–33822. 10.18632/oncotarget.5668 26393880 PMC4741804

[71] Wilczynska A, Bushell M. The complexity of miRNA-mediated repression. Cell Death Differ. 2015;22: 22–33. 10.1038/cdd.2014.112 25190144 PMC4262769

[72] Dalla Venezia N, Vincent A, Marcel V, Catez F, Diaz J-J, Dalla Venezia N, et al Emerging Role of Eukaryote Ribosomes in Translational Control. Int J Mol Sci. Multidisciplinary Digital Publishing Institute; 2019;20: 1226 10.3390/ijms20051226 30862090 PMC6429320

