# Supplementary figures and images for: MicroRNA modulated networks of adaptive and innate immune response in pancreatic ductal adenocarcinoma
Source: PLoS One. 2019 May 31;14(5):e0217421. doi: 10.1371/journal.pone.0217421 (PMC6544344; doi:10.1371/journal.pone.0217421)

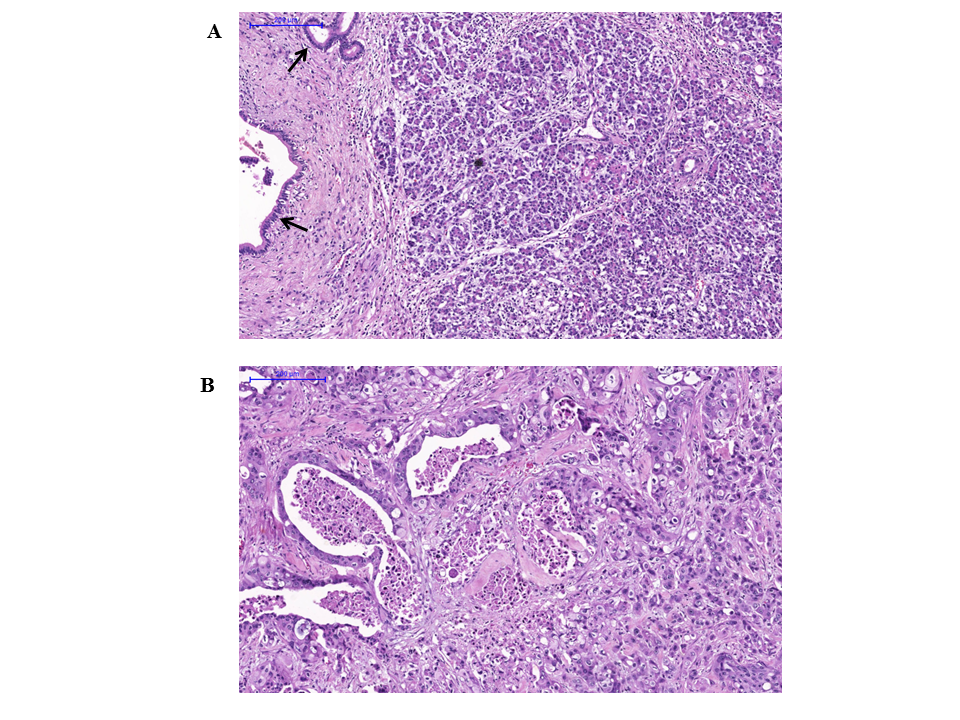

Supplement: S1 Fig — Representative example of macrodissected areas: Hematoxylin and Eosin (H&E) stained section showing in (A) adjacent normal tissue; normal pancreatic ducts are shown by the arrows and (B) pancreatic ductal adenocarcinoma cells and stromal component. Magnification: 200 μm. (TIF) [file pone.0217421.s001.tif]

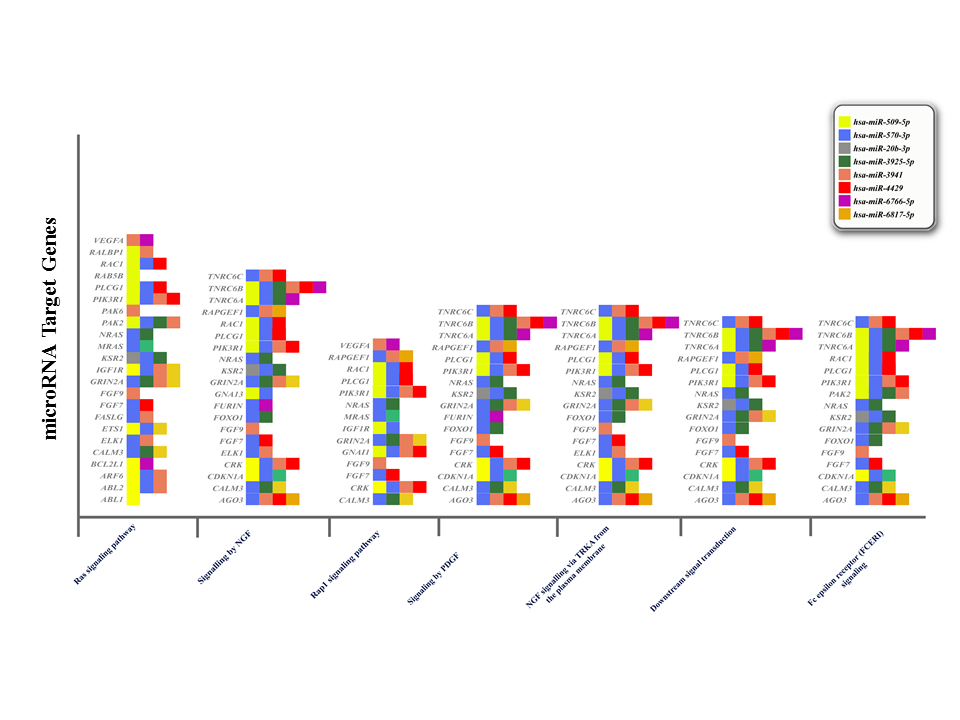

Supplement: S3 Fig — Graphical representation of miRNA target genes (Y-axis) according to significantly enriched pathways (p<0.01) (X-axis). (TIF) [file pone.0217421.s003.TIF]
